# Real-world use of theophylline in critically ill patients with sinus pauses: a case series

**DOI:** 10.3389/fcvm.2026.1731993

**Published:** 2026-02-16

**Authors:** Muhammad Ali Elahi, Hamza Jalal, William Frick, Bryan D. Kraft

**Affiliations:** Department of Medicine, Washington University School of Medicine, St. Louis, MO, United States

**Keywords:** bradyarrhythmia, clinical electrophysiology, pharmacotherapy, sinus pause, theophylline

## Abstract

**Background:**

A sinus pause is is defined as a delay in sinus node depolarization exceeding 3 s following atrial depolarization. In patients with pauses greater than 5 s accompanied by hemodynamic compromise, the primary treatment is permanent pacemaker (PPM) implantation. However, in the presence of an active infection, alternative strategies such as temporary transvenous pacing or pharmacologic agents may be more appropriate. This report highlights the use of theophylline as a non-invasive therapeutic option in two patients with asymptomatic sinus pauses who had contraindications to PPM placement.

**Cases:**

Patient A was a 74-year-old woman with a history of pulmonary arterial hypertension treated with subcutaneous treprostinil and a recent episode of catheter-associated *Staphylococcus aureus* bacteremia, for which she was receiving chronic suppressive doxycycline. She was admitted for acute decompensated heart failure. Patient B was a 24-year-old man with a history of cerebral palsy and chronic respiratory failure who was admitted for bacterial pneumonia. While in the intensive care unit, both patients experienced recurrent, asymptomatic sinus pauses lasting up to 8.6 s.

**Discussion:**

Given their active infections, both patients were deemed poor candidates for device placement and were therefore initiated on theophylline. Within 24 h of initiation, the sinus pauses resolved in both patients. Theophylline doses were adjusted to maintain a therapeutic concentration between 10 and 20 µg/mL. Later during hospitalization, Patient A developed atrial fibrillation with rapid ventricular response, prompting discontinuation of theophylline. Patient B was discharged on theophylline 50 mg twice daily, along with a 30-day event monitor and cardiology follow-up. Theophylline exerts its positive chronotropic effects primarily through non-selective phosphodiesterase inhibition and adenosine-1 receptor antagonism. These mechanisms enhance automaticity and reduce atrioventricular node refractoriness, augmenting sinus node automaticity and reducing the frequency and duration of sinus pauses.

**Conclusion:**

While these cases highlight the potential role of theophylline as a temporizing pacing strategy, additional research is needed to determine whether and how theophylline can be safely incorporated into treatment protocols for patients who are not suitable for device-based pacing.

## Introduction

The incidence of clinically significant sinus pauses or sinus node dysfunction is approximately 2% among ICU patients (1). A sinus pause is characterized by a delay in sinus node depolarization exceeding 3 s following atrial depolarization and typically reflects underlying sinus node dysfunction. Etiologies can be intrinsic, such as age-related degenerative fibrosis, structural cardiac remodeling, or genetic channelopathies, or extrinsic, such as pharmacologic agents, metabolic disturbances, heightened vagal tone, and infections (2, 3). The clinical relevance of a sinus pause is determined by both its duration and the presence of associated symptoms, such as palpitations, dizziness, or syncope. In patients with pauses greater than 5 s accompanied by hemodynamic compromise, the primary treatment is permanent pacemaker (PPM) implantation, along with correction of any reversible contributors (3). However, management becomes more complex in the presence of an active infection, where the risk of device implantation is heightened. In such scenarios, alternative strategies may be more appropriate, such as temporary transvenous pacing, leadless pacemakers, or pharmacologic agents (2, 4–6). This study describes a real-world experience using theophylline as a non-invasive therapeutic option in two patients with asymptomatic sinus pauses who had contraindications to PPM placement.

## Case 1: Description and diagnostic assessment

Patient A was a 74-year-old woman with a past medical history of chronic obstructive pulmonary disease, chronic hypoxemic respiratory failure requiring 10 L/min of supplemental oxygen at home, obstructive sleep apnea treated with nightly bilevel positive airway pressure, pulmonary arterial hypertension (PAH) managed with subcutaneous treprostinil, and a recent episode of central venous catheter-associated methicillin-resistant *Staphylococcus aureus* (MRSA) bacteremia for which she was receiving chronic suppressive doxycycline. She was admitted for acute decompensated heart failure. The PAH of the patient was managed in the outpatient setting with a continuous subcutaneous treprostinil infusion, titrated to a maintenance dose of 80 ng/kg/min. The most recent transthoracic echocardiogram (TTE) prior to admission showed a left ventricular ejection fraction of 70% with no right ventricular (RV) enlargement and normal RV function. On admission, repeat TTE was concerning and revealed marked RV enlargement, with a right ventricular internal dimension in diastole of 5.5 cm, along with concern for worsening RV function. These findings were believed to be secondary to inadequate absorption of treprostinil.

**Figure 1:**
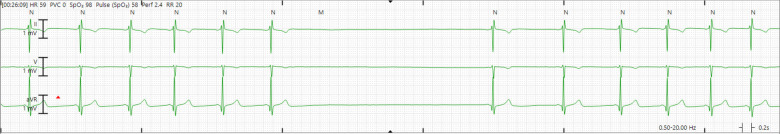
Telemetry strip demonstrating a sinus pause of 4.2 s (patient A).

**Figure 2:**
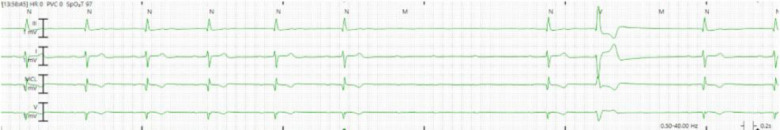
Telemetry strip demonstrating variable and advanced atrioventricular block (patient A).

On hospital day 9, telemetry monitoring revealed an 8.6-s sinus pause (Figure 1). The patient remained asymptomatic and hemodynamically stable. However, telemetry also raised concern for variable and advanced atrioventricular (AV) block (Figure 2). In light of this new conduction abnormality, she was transferred to the medical intensive care unit for closer cardiac monitoring. The differential diagnosis for the sinus pauses included intrinsic degenerative sinus node disease, ischemia, medication-induced bradyarrhythmia, metabolic abnormalities, and infection-related involvement of the conduction system. Of note, serum electrolytes remained within normal limits during this period, and no atrioventricular nodal-blocking agents were initiated prior to the rhythm disturbances. Thus, the current infection in conjunction with intrinsic degenerative sinus node dysfunction was thought to be the most likely etiology.

Given her recent history of MRSA bacteremia, she was considered at high risk for infectious complications associated with device placement. After consultation with cardiology colleagues, she was initiated on theophylline at a dose of 150 mg every 12 h. Her sinus pauses and bradycardia resolved. Based on serum trough levels, her theophylline dose was subsequently increased to 300 mg every 12 h to maintain a therapeutic range of 10–20 µg/mL.

However, on hospital day 21, the patient developed increasing lightheadedness, fatigue, and hypotension in the setting of sinus bradycardia. She was treated with atropine, which increased her heart rate up to 120 bpm. Shortly thereafter, she converted to atrial fibrillation with rapid ventricular response, prompting discontinuation of theophylline.

Following this episode, bradycardic episodes were asymptomatic and occurred only during sleep, with no further pauses. She was discharged with atrial fibrillation with a 30-day event monitor and a plan for elective cardioversion.

## Case 2: Description and diagnostic assessment

Patient B was a 24-year-old man with a history of cerebral palsy, tracheostomy, gastrostomy tube dependence, and chronic respiratory failure requiring 10 L/min of supplemental oxygen and nocturnal non-invasive positive pressure ventilation (NIPPV) at home. He was hospitalized for acute-on-chronic hypoxemic respiratory failure secondary to COVID-19 with superimposed bacterial pneumonia. On admission, he had sinus tachycardia (heart rate 100–110 bpm) and required supplemental 10 L/min of oxygen via tracheostomy collar. Broad-spectrum antibiotics were initiated for the bacterial pneumonia, and remdesivir and dexamethasone were started for severe COVID-19. Polymerase chain reaction testing of a tracheal aspirate for pneumonia pathogens identified *Streptococcus pneumoniae*, extended-spectrum beta-lactamase-producing organisms, MRSA, *Pseudomonas aeruginosa*, and *Moraxella catarrhalis*. Blood cultures and urinalysis were negative.

**Figure 3:**
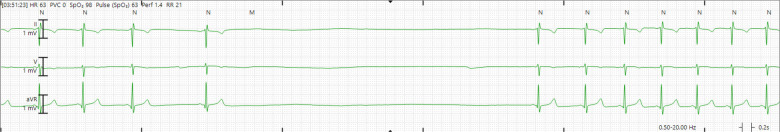
Telemetry strip demonstrating a sinus pause of 6.6 s (patient B).

On hospital day 9, telemetry revealed multiple episodes of bradycardia, with heart rates in the 40's, as well as sinus pauses lasting up to 7 s, all occurring without junctional or ventricular escape (Figure 3). Blood pressure and oxygen saturation remained stable. These pauses were initially attributed to high vagal tone, possible dislodgement of the gastrostomy tube, or the absence of home NIPPV. After consultation with cardiology colleagues, and in the absence of compelling symptoms, the patient was managed without immediate intervention.

On hospital day 11, the patient experienced multiple asymptomatic sinus pauses lasting up to 5 s. He was afebrile, and blood cultures showed no growth. Serum electrolytes remained within normal limits. Transthoracic echocardiography demonstrated a normal left ventricular ejection fraction of 62%. Given these findings, the sinus pauses were attributed to active sepsis. Although the patient did not exhibit hemodynamic compromise at the time, the duration and frequency of sinus pauses prompted concern among the ICU team. As a result, the decision was made to initiate theophylline therapy to prevent the risk of potential future hemodynamic compromise. On hospital day 13, the patient was initiated on theophylline at a dose of 100 mg every 12 h due to persistent pauses and ineligibility for a pacemaker or temporary pacing in the presence of active sepsis. Within 24 h of initiation, the sinus pauses resolved. Theophylline levels measured on days 15 and 17 were subtherapeutic at 7.3 and 7.6 µg/mL, respectively (target therapeutic range 10–20 µg/mL; Table 1). On day 15, intermittent bradycardia with heart rates in the 30's to 40's was noted without documented sinus pauses; by day 17, sinus pauses had ceased entirely. On hospital day 19, the patient developed multiple episodes of sinus tachycardia, with heart rates in the 130–140 s. Given the persistent tachycardia, theophylline was held and subsequently restarted at a reduced dose of 50 mg twice daily on day 21. During the final 72 h of hospitalization, no further episodes of bradycardia or sinus pauses were observed.

**Table 1:** Daily heart rates (mean ± SD) in relation to serum theophylline levels.

Patient A		Patient B	
Theophylline (µg/mL)	Heart rate (bpm)	Theophylline (µg/mL)	Heart rate (bpm)
8.0	70 ± 5	7.3	76 ± 14
8.7	83 ± 12	7.6	86 ± 20
14.1	92 ± 20	6.9	93 ± 22
7.7	100 ± 9		

bpm, beats per minute; SD, standard deviation.

The patient was discharged on theophylline 50 mg every 12 h, a 30-day event monitor, and outpatient cardiology follow-up. Theophylline was then discontinued at his 1-month outpatient follow-up.

## Discussion

Herein, we report two cases that illustrate real-world experience with the use of theophylline for the treatment of sinus pauses in hospitalized patients. Specifically, both cases involved critically ill patients with a time-limited course of sinus node dysfunction attributable to extrinsic factors. Theophylline exerts its positive chronotropic effects primarily through non-selective inhibition of phosphodiesterase and antagonism of adenosine (A1) receptors in the sinus and AV nodes. By blocking adenosine receptors, theophylline enhances automaticity and reduces AV node refractoriness. Antagonism of adenosine also increases cyclic adenosine monophosphate levels, leading to increased calcium influx and promotion of depolarization. Through these actions, theophylline augments sinus node automaticity and reduces the frequency and duration of sinus pauses (2, 3, 7). However, theophylline does not improve conduction in infranodal block, as its principal actions are located at the adenosine (A1) receptors, which are more densely expressed in the sinus and AV nodes than in the infranodal conduction system (8). Importantly, theophylline may serve as a non-invasive alternative or temporizing strategy when device implantation is contraindicated by active infection or prohibitive procedural risk (2, 3).

Theophylline has significant limitations. Notably, it has a narrow therapeutic index and significant proarrhythmic potential, which often limits its use in high-risk or older patient populations (9). In both of our patients, theophylline dosing was intentionally conservative, reflecting concerns regarding its narrow therapeutic window and risk of arrhythmia. Initial doses were selected to minimize adverse effects. Although theophylline helped resolve the sinus pauses, it was also associated with tachycardia, necessitating dose reduction or discontinuation despite serum drug levels remaining subtherapeutic. We did not observe a clear dose–response relationship between theophylline levels and patient heart rate, as shown in Table 1, although heart rate in the ICU can be confounded by many other clinical variables, such as patient stress level, volume status, and infection severity. While traditional therapeutic ranges (10–20 µg/mL) are derived largely from pulmonary indications, lower serum concentrations may be sufficient to produce sufficient chronotropic effects. These observations suggest that typical starting doses of 100 mg twice daily may not be necessary, and lower doses should be considered first. We cannot rule out other potential confounders, although we did not observe significant electrolyte abnormalities, medication changes, or worsening infection after initiation of theophylline. This was consistent across both patients and strengthens the association between the initiation of theophylline and rhythm improvement.

Prior studies have evaluated the efficacy of theophylline in treating bradyarrhythmias. In elderly hospitalized patients with symptomatic sinus bradycardia, oral theophylline increased resting heart rate and abolished sinus pauses of ≥2.5 s (10). The THEOPACE randomized controlled trial found that for hospitalized patients with sick sinus syndrome, theophylline was comparable to pacemaker therapy in improving subjective symptom scores. Although both theophylline and pacemaker implantation reduced the incidence of syncopal events, syncopal events occurred less frequently in patients who received pacemaker therapy compared with those treated with theophylline (11). Furthermore, in a propensity-score controlled study, theophylline administration was shown to effectively prevent recurrent syncopal episodes in patients with unexplained syncope without prodrome and normal ECGs. The authors noted that a significant benefit in a subgroup of patients with syncope due to asystolic atrioventricular block, reporting a relative risk reduction of 87% (12).

Contemporary data on the use of theophylline as a pacing strategy in selected patients remain limited. Prior studies have suggested benefit in older patients for whom device implantation is contraindicated (5, 6). A recent case report documented the successful use of theophylline to correct sinus node dysfunction in the setting of acute-on-chronic lithium toxicity, in which traditional pacing strategies posed challenges due to patient preference and clinical complexity (13). This report and ours suggest that theophylline treatment for sinus node dysfunction may be applicable across a broad range of clinical scenarios. Alternative strategies during active infections, such as temporary transvenous pacing, leadless pacemakers, or epicardial devices, are better described in contemporary literature. These cases suggest that theophylline can provide temporary chronotropic support in the setting of extrinsic sinus node dysfunction due to an acute illness; however, there is a risk of inducing tachyarrhythmias.

Prospective studies comparing theophylline with alternative pacing strategies for extrinsic, asymptomatic prolonged sinus pauses in critically ill patients are needed to better define its efficacy and safety. Optimal dosing, therapeutic drug monitoring, treatment duration, and identification of responsive patient subgroups remain poorly defined and warrant further investigation, particularly in patients for whom device-based pacing is not suitable. While long-term use of theophylline in such scenarios is unlikely to be necessary, given the potential for theophylline-induced adverse cardiac effects, the short- and intermediate-term safety profiles in such patients should be further studied. While these cases highlight the potential role of theophylline as a temporizing pacing strategy, additional research is needed to determine whether theophylline can be incorporated into algorithmic treatment protocols for short-term use in critically ill patients who are not suitable for device-based pacing.

## Patient perspective

Informed, written consent from the legal representatives of both patients was obtained prior to this case series.

## Data Availability

The raw data supporting the conclusions of this article will be made available by the authors, without undue reservation.
